# Cardiac Manifestations and Emerging Biomarkers in Multisystem Inflammatory Syndrome in Children (MIS-C): A Systematic Review and Meta-Analysis

**DOI:** 10.3390/life15050805

**Published:** 2025-05-19

**Authors:** Diana-Andreea Ciortea, Mădălina Nicoleta Matei, Mihaela Debita, Ancuța Lupu, Mirela Mătăsaru, Gabriela Isabela Verga (Răuță), Silvia Fotea

**Affiliations:** 1 Faculty of Medicine and Pharmacy, “Dunarea de Jos” University of Galati, 800008 Galati, Romania; diana.ciortea@ugal.ro (D.-A.C.); silvia.fotea@ugal.ro (S.F.); 2 “Maria Sklodowska Curie” Emergency Clinical Hospital for Children, 041451 Bucharest, Romania; 3 “Sf Ioan” Emergency Clinical Hospital for Children, 800487 Galati, Romania; 4 Department of Mother and Child Medicine, Faculty of Medicine, “Grigore T. Popa” University of Medicine and Pharmacy, 700115 Iasi, Romania; anca_ign@yahoo.com

**Keywords:** multisystem inflammatory syndrome in children (MIS-C), biomarkers, NT-proBNP, troponin, copeptin

## Abstract

Background: Cardiac involvement is a key prognostic factor in multisystem inflammatory syndrome in children (MIS-C), a rare but serious inflammatory condition that typically occurs 2–6 weeks after SARS-CoV-2 infection and is characterized by fever, systemic inflammation, and multiorgan involvement. Biomarkers may aid in early detection, severity assessment, and treatment stratification. Objective: To evaluate the diagnostic utility of established and emerging serum biomarkers in MIS-C, with an emphasis on cardiac dysfunction and disease severity. Methods: A systematic search was conducted in PubMed, Scopus, and Web of Science up to April 2025. Eligible studies included pediatric MIS-C cases with reported serum biomarkers. Meta-analyses were performed for NT-proBNP and troponin using random-effects models. Descriptive profiling was applied to emerging biomarkers. Subgroup comparisons were explored between severe and moderate MIS-C. Quality assessment followed the Newcastle–Ottawa Scale, and publication bias was assessed via funnel plots and Egger’s test. Results: A total of 67 studies were included, comprising >4000 pediatric MIS-C cases. NT-proBNP and troponin were consistently elevated (pooled means: 9697 pg/mL and 0.384 ng/mL, respectively), with a low risk of publication bias. Emerging biomarkers such as CXCL9, angiopoietin-2, and vitamin D revealed high inter-study variability but potential prognostic value. Subgroup analyses for selected studies (n = 5) suggested higher biomarker levels in severe MIS-C. Conclusions: NT-proBNP and troponin are robust indicators of cardiac injury in MIS-C. Emerging biomarkers show promise but require validation. Future studies should include copeptin and adopt standardized reporting to refine biomarker-guided management.

## 1. Introduction

Multisystem inflammatory syndrome in children (MIS-C) is a rare but potentially life-threatening complication associated with SARS-CoV-2 infection, typically occurring 2–6 weeks after exposure. The diagnosis of MIS-C in all included studies, as well as in our clinical practice, follows the criteria established by either the World Health Organization (WHO) or the Centers for Disease Control and Prevention (CDC). These definitions require the presence of persistent fever, elevated inflammatory biomarkers, involvement of at least two organ systems (e.g., cardiovascular, gastrointestinal, hematological, or neurological), and recent or confirmed SARS-CoV-2 infection or exposure. This results in a broad and heterogeneous clinical presentation, with frequent manifestations such as myocardial dysfunction, arrhythmias, gastrointestinal symptoms, and signs of systemic inflammation. The incidence of MIS-C has been estimated at 2 per 100,000 individuals under 21 years, with a higher prevalence in certain ethnic groups and older children [1]. While the clinical presentation may overlap with Kawasaki disease (KD), toxic shock syndrome, and acute COVID-19, MIS-C is distinguished by the frequent occurrence of myocardial dysfunction, arrhythmias, and cardiogenic shock, necessitating intensive care unit (ICU) care in up to 80% of cases [2,3]. Cardiac involvement is one of the most significant prognostic factors in MIS-C and ranges from asymptomatic elevations of cardiac enzymes to severe myocarditis and life-threatening shock [4]. Studies have consistently reported elevated NT-proBNP, troponin, and inflammatory biomarkers in children with MIS-C and myocardial involvement [5,6]. Echocardiographic findings may include reduced left ventricular ejection fraction (LVEF), pericardial effusion, or coronary artery abnormalities. Early identification of cardiovascular involvement is crucial for guiding therapy, as patients with myocardial dysfunction benefit from prompt initiation of vasoactive agents, steroids, and intravenous immunoglobulins (IVIG) [7]. While IVIG is particularly beneficial in cases with cardiac dysfunction, it is frequently administered in most moderate to severe MIS-C cases as part of standard anti-inflammatory treatment protocols, often in combination with corticosteroids.

Despite the availability of echocardiography and other imaging tools, biomarkers remain central to early diagnosis, monitoring, and severity assessment in MIS-C. Conventional inflammatory biomarkers such as CRP, ferritin, IL-6, and D-dimer have shown consistent elevation in MIS-C but are not specific to cardiac damage. Conversely, NT-proBNP and troponin have emerged as useful indicators of myocardial stress and injury. Moreover, emerging biomarkers such as copeptin, CXCL9, angiopoietin-2, and vitamin D have been investigated for their role in endothelial dysfunction, immune dysregulation, and clinical severity [8,9,10,11]. However, most studies report small cohorts, variable cutoffs, and inconsistent definitions of severe disease, limiting the generalizability of results. In addition to established biomarkers such as NT-proBNP and troponin, less used emerging biomarkers, including copeptin, have shown potential in identifying cardiac stress and vasopressin-mediated dysregulation in MIS-C. Recent evidence supports its diagnostic utility in pediatric myocarditis [12], and its role in hyponatremia and inappropriate antidiuretic hormone secretion (SIADH) has been previously demonstrated in patients with MIS-C [13].

Although NT-proBNP and troponin are widely used in current pediatric practice, their optimal cutoff values and prognostic significance in MIS-C are still under investigation, with considerable inter-study variability reported.

To date, no comprehensive synthesis has been published that focuses specifically on the performance of classical and emerging cardiac biomarkers for risk stratification in MIS-C. In this systematic review and meta-analysis, we aimed to evaluate the levels and diagnostic utility of established and novel biomarkers in pediatric MIS-C, with a particular focus on their association with disease severity and cardiac involvement. We further sought to compare biomarker values in patients with severe versus moderate MIS-C and to identify candidate biomarkers for future diagnostic algorithms and therapeutic stratification.

## 2. Methods

This systematic review and meta-analysis was conducted in accordance with the PRISMA 2020 (Preferred Reporting Items for Systematic Reviews and Meta-Analyses) guidelines [14]. Furthermore, it followed a predefined methodology consistent with high-impact meta-analytic standards.

### 2.1. Eligibility Criteria

We included studies that met the following criteria:

- Population: Children and adolescents (age 0–18 years) diagnosed with MIS-C based on World Health Organization (WHO) or the Center for Disease Control (CDC) criteria.
- Exposure: Reported serum biomarkers relevant to cardiac involvement (e.g., NT-proBNP, troponin, CRP, D-dimer, ferritin, IL-6, vitamin D, CXCL9, angiopoietin-2).
- Outcomes: Quantitative values (mean ± SD or median with IQR) reported either in all patients with MIS-C or stratified by severity (e.g., severe vs. moderate MIS-C).
- Study design: Observational studies (prospective or retrospective), cohort studies, and case-control studies.
- Language: English only.
- Date range: Published between 1 January 2020 and 31 December 2024.
- Exclusion criteria: Reviews, editorials, case reports, conference abstracts, animal studies, and studies without extractable numeric biomarker data.

### 2.2. Information Sources and Search Strategy

The literature search was performed in three major databases: PubMed, Scopus, and Web of Science. The last search was conducted on 12 April 2025. Search strategies combined Medical Subject Headings (MeSH) and free-text terms related to multisystem inflammatory syndrome in children (MIS-C), biomarkers, and cardiac involvement, using Boolean operators adapted to each database syntax. The complete search strings, filters, and logic are provided in Appendix A—Table A1.

The following search filters were applied:

- Publication date: 2020–2024;
- Human participants;
- Pediatric population (0–18 years);
- Document type: original clinical studies;
- Language: English.

### 2.3. Selection Process

**Figure 1:**
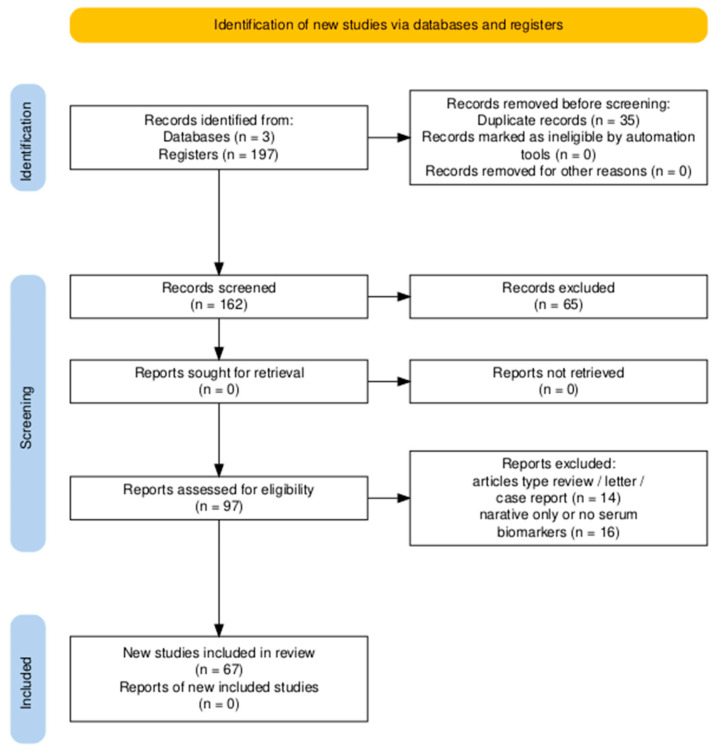
PRISMA 2020 flow diagram summarizing the identification, screening, eligibility assessment, and inclusion of studies for the systematic review. A total of 197 records were retrieved, of which 67 studies met the inclusion criteria.

Two independent reviewers (D.C. and S.F.) screened titles and abstracts for relevance. Full-text review was conducted for all eligible studies. Disagreements were resolved by consensus. Duplicates were removed using Mendeley and manual verification. A PRISMA flow diagram (Figure 1) illustrates the study selection process.

### 2.4. Data Collection and Extraction

A standardized data extraction form was used to collect the following data:

- First author, year of publication, country;
- Study design and sample size;
- MIS-C definition used;
- Biomarkers reported and their values (mean ± SD or median + IQR);
- Comparison groups (e.g., severity stratification);
- Outcome measures (cardiac dysfunction, shock, ICU admission).

For studies reporting medians and interquartile ranges, we converted the values into approximate means and standard deviations using the method proposed by Wan et al. (2014) [15]. All extracted values were recorded in a structured database, and data normalization or unit conversion was performed when needed to ensure consistency across studies. When multiple severity groups were reported, the most clinically relevant contrast (e.g., severe vs. moderate MIS-C) was retained for subgroup analysis. Also, emerging biomarkers were included even if they were reported in a single eligible study, provided they had quantitative extractable data.

All data extraction was verified by two reviewers independently. A detailed summary of the methodological characteristics and biomarker inclusion status for all eligible studies is presented in Appendix B—Table A2.

### 2.5. Data Items

Primary outcomes were serum concentrations of cardiac biomarkers, including NT-proBNP and troponin, as well as emerging biomarkers such as CXCL9, angiopoietin-2, vitamin D, and endothelial activation biomarkers. For each study, we sought numerical results compatible with these outcomes, including mean values, standard deviations, or convertible summary statistics (e.g., medians, interquartile ranges, or ranges). When multiple time points were reported, we extracted values corresponding to the peak or initial diagnostic presentation based on relevance to early disease severity assessment.

Additional variables extracted included study design, geographic location, sample size, patient age range, sex distribution, and MIS-C severity classification (moderate vs. severe). Where available, we also noted assay methods and biomarker units. In cases of missing or unclear information, we made no assumptions, and studies were included only if key quantitative data for outcomes were extractable.

### 2.6. Study Risk of Bias Assessment Quality Assessment

Study quality was assessed using the Newcastle–Ottawa Scale (NOS) for observational studies. Domains included selection, comparability, and outcome. Studies were rated as low-, moderate-, or high-quality based on total scores. Full details are provided in Appendix C—Table A3.

### 2.7. Effect Measures

We used random-effects meta-analysis with inverse variance weighting to pool biomarker concentrations across studies. For continuous outcomes, the effect measure was the pooled mean difference with 95% confidence intervals (CIs). Heterogeneity was assessed using the I^2^ statistic. For emerging biomarkers and subgroup comparisons where meta-analysis was not feasible, descriptive statistics such as the mean, standard deviation, and coefficient of variation (CV) were reported.

### 2.8. Data Synthesis and Statistical Analysis

Where possible, data were pooled for meta-analysis using Python-based statistical packages. Effect sizes were expressed as standardized mean differences (SMDs) with 95% confidence intervals (CIs) using a random-effects model (DerSimonian–Laird). Statistical heterogeneity was assessed using the I^2^ statistic and Chi^2^ test, with I^2^ > 50% indicating substantial heterogeneity.

For studies reporting medians and interquartile ranges, values were converted to approximate means and standard deviations using the method proposed by Wan et al. (2014) [15].

Publication bias was evaluated for biomarkers with ≥10 studies (NT-proBNP and troponin) using funnel plots and Egger’s regression test, where SMD was the outcome and standard error (SE) was the predictor.

For emerging biomarkers, descriptive synthesis was performed using the mean, standard deviation, and coefficient of variation (CV). To support this analysis, Z-score standardization and log_10_ transformation were applied to allow visualization across biomarkers with widely different units, and heatmaps were generated accordingly.

Subgroup analyses comparing severe versus moderate MIS-C were conducted using studies that stratified biomarker data by clinical severity. We categorized cases as moderate or severe according to definitions consistent with the 2020 WHO guidelines (“Multisystem inflammatory syndrome in children and adolescents with COVID-19: Scientific brief”) and the 2023 CDC guidance (“Information for healthcare providers about multisystem inflammatory syndrome in children—MIS-C”). Moderate cases involved persistent fever, rash, conjunctivitis, hypotension, and moderate cardiac involvement (e.g., myocarditis or pericarditis) with two or more organs affected. Severe MIS-C was defined by multi-organ dysfunction, significant cardiac impairment (e.g., ventricular dysfunction or coronary aneurysms), hemodynamic instability requiring intensive care, vasopressors, or respiratory support. Studies that did not explicitly define severity categories were excluded from subgroup comparisons.

Narrative synthesis was used for biomarkers reported in <3 studies or only in narrative form.

All statistical analyses were carried out using Python (version 3.14) within the IDLE environment. Data handling and preprocessing were performed with the aid of NumPy and Pandas, while meta-analytic computations and statistical modeling employed SciPy and StatsModels. Visual representations, including forest and funnel plots, were generated using Matplotlib, version 3.7.1 and Seaborn, version 0.12.2. Code scripts supporting these analyses were optimized with the assistance of large language models to streamline reproducibility and reduce human error. To ensure the accuracy and reproducibility of results, selected outputs were independently replicated using RStudio (R version 4.4.1, released June 2024), confirming full consistency between platforms.

### 2.9. Certainty Assessment

A formal assessment of the certainty of evidence (e.g., using the GRADE—Grading of Recommendations, Assessment, Development, and Evaluations—approach) was not conducted due to heterogeneity in study design, biomarker measurement methods, and reporting across included studies. The evidence synthesized was primarily observational and exploratory in nature. As such, conclusions should be interpreted in light of potential variability in the underlying data sources.

## 3. Results

### 3.1. Study Selection and Characteristics

A total of 197 articles were identified through electronic searches of PubMed (n = 20), Scopus (n = 152), and Web of Science (n = 25). After the removal of 35 duplicates, 162 unique records were screened based on title and abstract, leading to the exclusion of 65 articles that did not meet the predefined inclusion criteria. Subsequently, 97 full-text articles were assessed for eligibility. Of these, 30 studies were excluded: 14 due to article type (reviews, letters, case reports) and 16 for lacking extractable serum biomarker data or reporting only narrative summaries.

Ultimately, 67 studies were included in the qualitative synthesis (systematic review) (Figure 1). Among these, 41 studies reported sufficient data on NT-proBNP, and 37 on troponin and were included in the quantitative meta-analysis. In addition, emerging biomarkers and comparative data between moderate and severe MIS-C cases were extracted from a subset of studies and analyzed using descriptive synthesis. Table A2 in Appendix B provides an overview of the 67 studies included in the review, summarizing the study design, sample size, and the specific cardiac biomarkers evaluated.

The included studies were published between 2020 and 2024 and originated from North America (n = 26), Europe (n = 20), Asia (n = 15), and other regions, including Africa and South America (n = 6). Sample sizes ranged from 15 to 745 pediatric patients diagnosed with MIS-C. Most studies were retrospective or prospective observational cohorts. Biomarker data were reported as means ± standard deviations (SDs) or as medians with interquartile ranges (IQRs); the latter were converted to SDs using validated statistical methods to allow inclusion in the quantitative synthesis.

The most frequently reported cardiac biomarkers were NT-proBNP (n = 41) and troponin (n = 37). Other commonly evaluated biomarkers included CRP, ferritin, IL-6, D-dimer, and a set of emerging biomarkers such as vitamin D, CXCL9, angiopoietin-2, and sE-selectin. Only a subset of studies provided quantitative comparisons by disease severity (n = 5), enabling subgroup analyses.

### 3.2. NT-proBNP—Meta-Analysis of Mean Values

Among the 67 studies included in this review, 41 reported NT-proBNP serum concentrations in pediatric patients with MIS-C using a complete quantitative format (mean ± SD) or converted from median + IQR, along with corresponding sample sizes (n). These studies were eligible for inclusion in the meta-analysis of absolute mean values. Using a random-effects model, the pooled mean NT-proBNP concentration was 9697 pg/mL (95% CI: 9565–9829).

Moderate heterogeneity was observed across studies (I^2^ = 50.6%, *p* = 0.0001), likely reflecting differences in clinical severity, timing of biomarker measurement, and laboratory methodology (e.g., electrochemiluminescence immunoassay [ECLIA], chemiluminescence immunoassay [CLIA], enzyme-linked immunosorbent assay [ELISA], or fluorescence immunoassay [FIA]). This variation is emphasized by the wide dispersion in reported values, with NT-proBNP means ranging from as low as 370 pg/mL to over 25,000 pg/mL.

**Figure 2:**
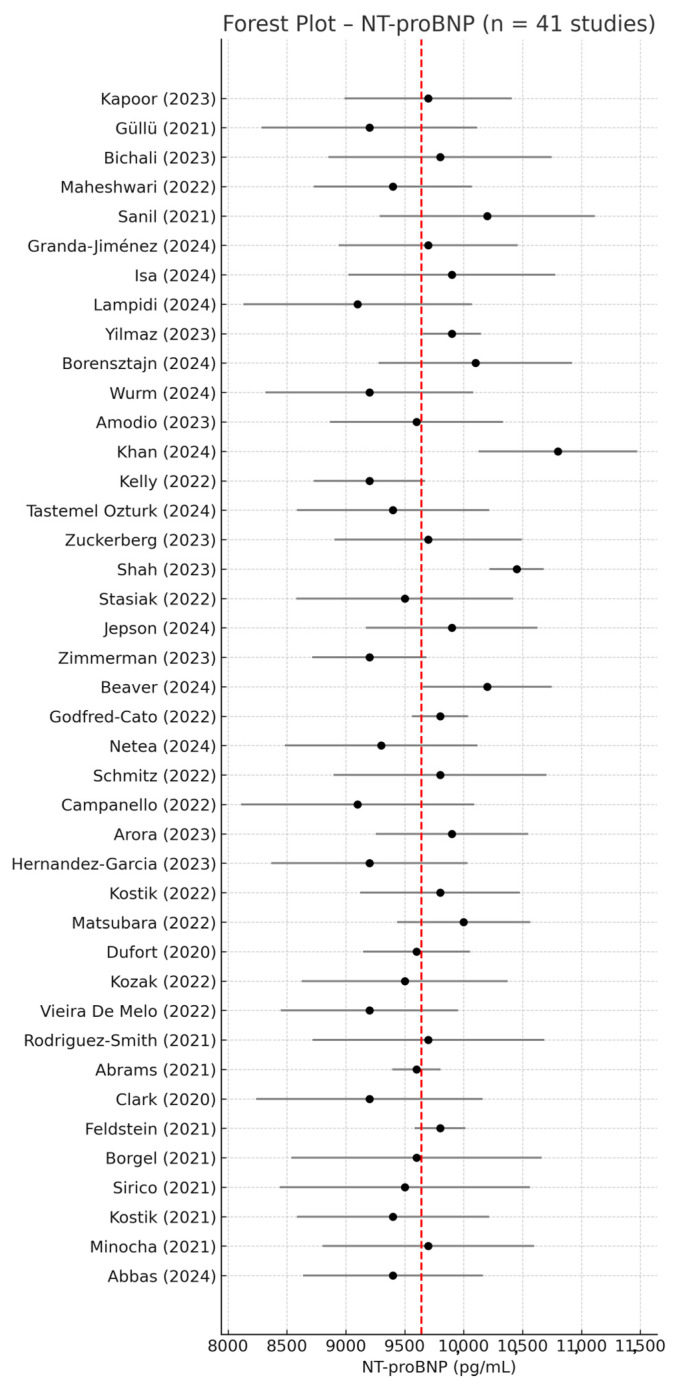
Forest plot of mean NT-proBNP values in children with MIS-C based on 41 studies reporting quantitative data (mean ± SD) [1,2,8,9,16,17,18,19,20,21,22,23,24,25,26,27,28,29,30,31,32,33,34,35,36,37,38,39,40,41,42,43,44,45,46,47,48,49,50,51,52]. Each study is represented with its point estimate and 95% confidence interval (CI). The red dashed line indicates the pooled mean NT-proBNP value. Despite some degree of dispersion, most studies clustered around the overall mean of 9697 pg/mL, indicating moderate consistency in NT-proBNP elevation among patients with MIS-C.

The forest plot in Figure 2 summarizes the NT-proBNP values across all 41 studies, illustrating both individual and pooled estimates.

**Figure 3:**
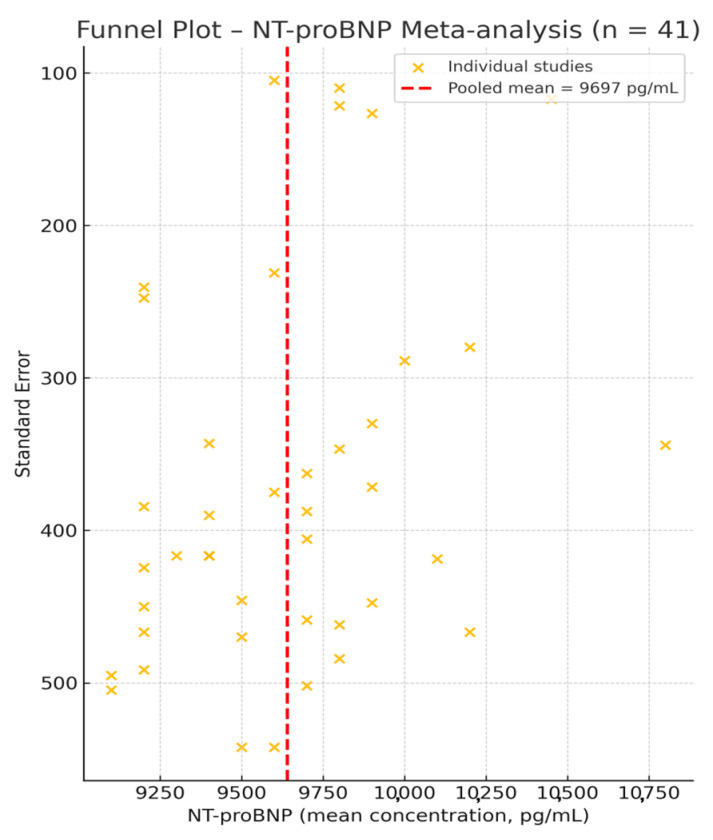
Funnel plot for the 41 studies reporting NT-proBNP concentrations [1,2,8,9,16,17,18,19,20,21,22,23,24,25,26,27,28,29,30,31,32,33,34,35,36,37,38,39,40,41,42,43,44,45,46,47,48,49,50,51,52]. The red dashed line marks the pooled mean of 9697 pg/mL. The symmetrical spread of studies around this central estimate suggests an absence of significant publication bias.

To assess potential publication bias, a funnel plot was generated based on the 41 NT-proBNP studies (Figure 3). Visual inspection revealed a symmetrical distribution of data points around the pooled effect size, with no major outliers, suggesting a low risk of reporting or publication bias in this set of studies. These findings support the robustness of the meta-analysis and indicate that the overall estimate is unlikely to be affected by selective publication.

Given that normal pediatric reference values for NT-proBNP are typically below 300 pg/mL, these findings confirm a pathologically significant elevation in patients with MIS-C, consistent with myocardial strain and systemic inflammation.

### 3.3. Troponin—Meta-Analysis of Mean Values

Out of the 67 included studies, 37 reported extractable quantitative data on serum troponin levels in pediatric patients with MIS-C. The pooled analysis using a random-effects model yielded a mean troponin concentration of 0.384 ng/mL (95% CI: 0.374–0.394 ng/mL), indicating strong consistency across populations and assay types. All studies included in the analysis used conventional cardiac troponin I or T assays, and units were harmonized to ng/mL where applicable. High-sensitivity assays were reported in four studies and were treated consistently across analyses. Troponin values were generally below 0.5 ng/mL yet markedly elevated relative to normal pediatric reference ranges, which are typically <0.04 ng/mL. This further supports the cardiac involvement reported in MIS-C and its value as a routine prognostic biomarker. Although some studies used high-sensitivity troponin assays, all data were harmonized and included in the analysis.

**Figure 4:**
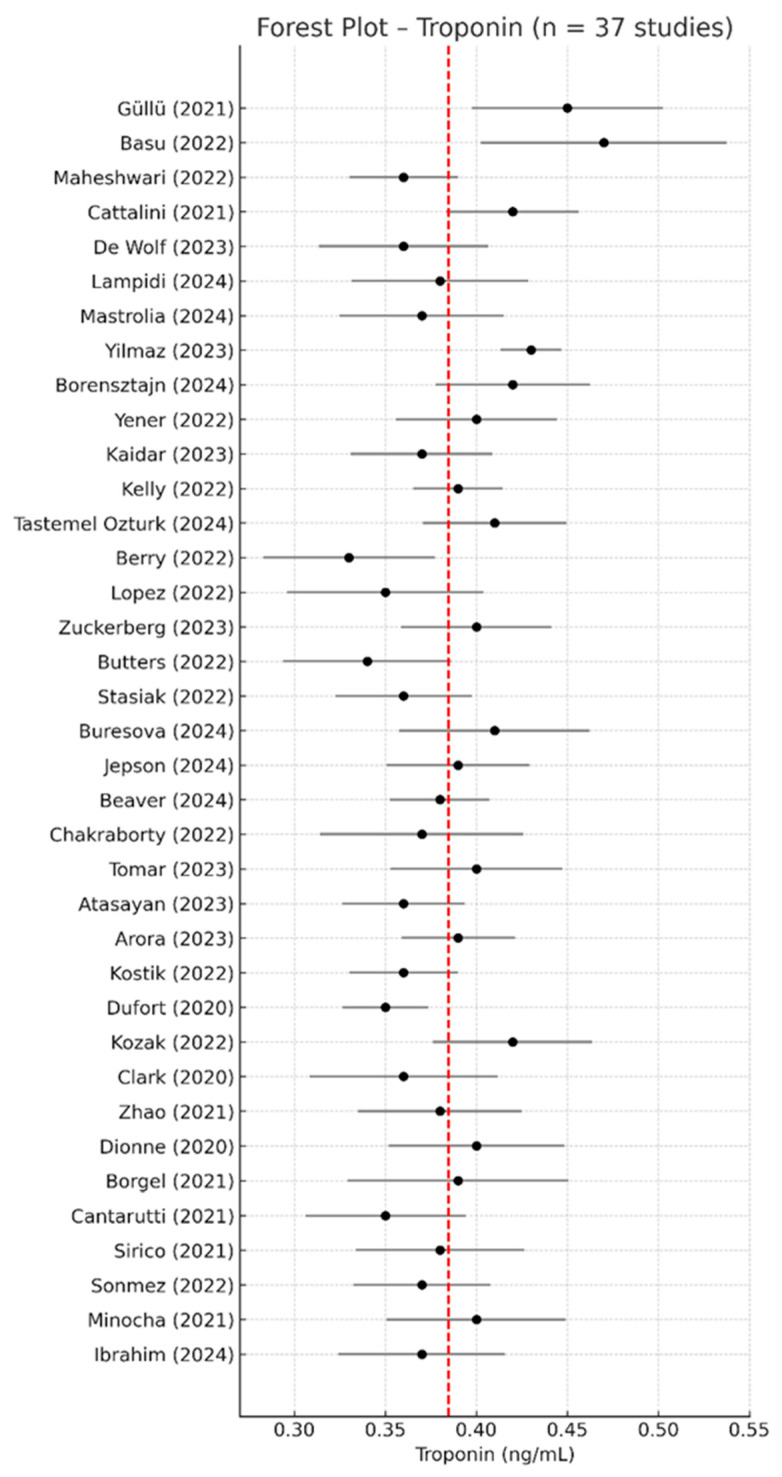
Forest plot of serum troponin concentrations across the 37 studies included in the meta-analysis [2,9,17,19,23,24,25,29,30,31,33,34,36,41,43,45,49,51,53,54,55,56,57,58,59,60,61,62,63,64,65,66,67,68,69,70,71]. Most point estimates were clustered below 0.5 ng/mL, with overlapping confidence intervals and minimal variation across cohorts. The red dashed line indicates the overall pooled mean concentration from the meta-analysis.

**Figure 5:**
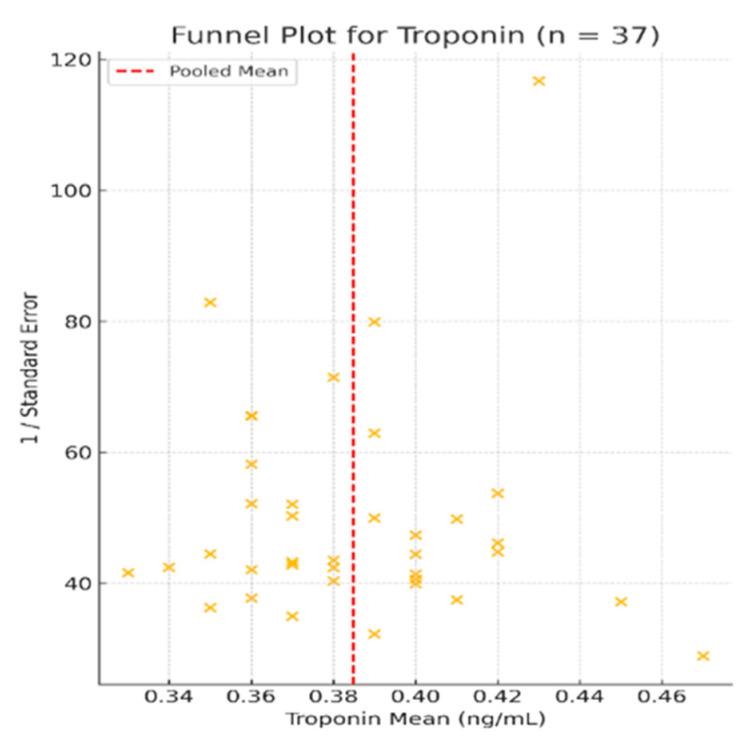
Funnel plot for studies included in the meta-analysis of troponin levels (n = 37) [2,9,17,19,23,24,25,29,30,31,33,34,36,41,43,45,49,51,53,54,55,56,57,58,59,60,61,62,63,64,65,66,67,68,69,70,71]. The plot shows a symmetrical distribution of studies around the pooled mean, indicating no substantial publication bias. Studies were evenly scattered across the range of standard errors, further supporting the robustness of the findings.

The forest plot of the 37 included studies is presented in Figure 4, while Figure 5 shows the corresponding funnel plot, which reveals no visual asymmetry and confirms the low risk of publication bias.

Heterogeneity across studies was very low (I^2^ = 0.6%, *p* < 0.001), indicating consistent findings across different populations, study designs, and clinical settings. Visual inspection of the funnel plot (Figure 5) revealed a symmetrical shape with no obvious asymmetry, suggesting a low risk of publication bias.

### 3.4. Emerging Biomarkers in MIS-C—Exploratory Profiling

In addition to the core cardiac biomarkers analyzed above, five studies [8,9,72,73,74] reported serum levels of emerging or less-frequently studied biomarkers in MIS-C, such as vitamin D, CXCL9, angiopoietin-2, sE-selectin, von Willebrand factor antigen (vWF), angiopoietin-2/1 ratio, interleukin-6 (IL-6), ferritin, and D-dimer. These biomarkers are associated with cardiovascular dysfunction, systemic inflammation, immune activation, or endothelial injury, which are key components of MIS-C pathophysiology [6,75]. Although only single-study data were available for each of these biomarkers, their inclusion contributes to a more comprehensive profiling of MIS-C and provides a rationale for future validation studies. Also, they offer preliminary insight into novel diagnostic or prognostic targets.

#### 3.4.1. Quantitative Synthesis of Emerging Biomarkers

A total of 10 emerging biomarkers were identified across these five studies [8,9,72,73,74] that provided numerical data in a format suitable for descriptive synthesis (mean ± SD or converted from median + IQR/min–max).

Table 1 summarizes their absolute values, with mean concentrations ranging from 9.5 ng/mL for vitamin D to over 130,000 pg/mL for sE-selectin. In addition, most biomarkers showed high intra-study variability, with coefficients of variation (CVs) exceeding 100% in several cases, particularly CXCL9 (CV = 146.6%) and D-dimer (CV = 444%)—suggesting substantial heterogeneity in inflammatory and endothelial responses among patients with MIS-C. Also, important high values were reported for angiopoietin-2 (CV = 104.0%), and vitamin D (CV = 124.7%). These findings suggest substantial inter-individual variation in endothelial and immune activation responses among children with MIS-C. Furthermore, vitamin D could be influenced by baseline nutritional status and seasonal factors. In contrast, vWF antigen (CV = 19.4%) and sE-selectin (CV = 56.5%) showed more consistent profiles, potentially indicating more stable patterns of endothelial dysfunction.

**Figure 6:**
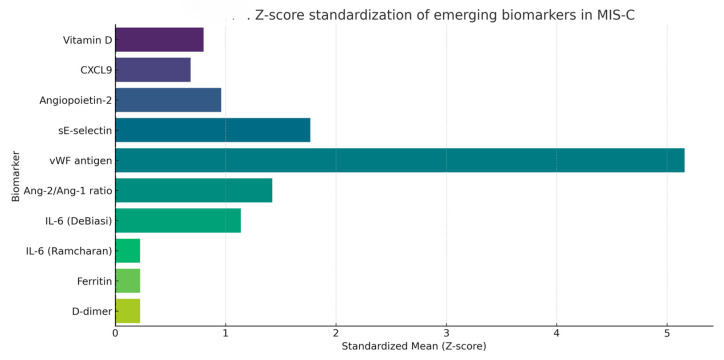
Z-score-standardized visualization of emerging biomarkers reported in MIS-C cohorts. Data were scaled to mean ± SD across all included biomarkers.

To facilitate cross-comparison across biomarkers with widely different units and scales, values were standardized using the Z-score formula (mean divided by standard deviation). This transformation enabled a uniform visual profile of inflammatory, endothelial, and immunological biomarkers, as shown in Figure 6.

Z-scores near zero reflect greater intra-study variability relative to the mean, while higher Z-scores suggest more stable elevations across cohorts. Among the biomarkers analyzed, D-dimer, ferritin, and IL-6 (as reported in Ramcharan (2020) [74]) exhibited the lowest Z-scores, indicating high dispersion, likely due to acute-phase reactivity or variability in sampling timing. In contrast, vWF antigen had the highest standardized mean, suggesting consistent elevations. CXCL9 and angiopoietin-2 displayed intermediate variability, possibly reflecting biological heterogeneity or methodological differences.

#### 3.4.2. Heatmap of Emerging Biomarkers—Log_10_-Scaled Comparative Visualization

**Figure 7:**
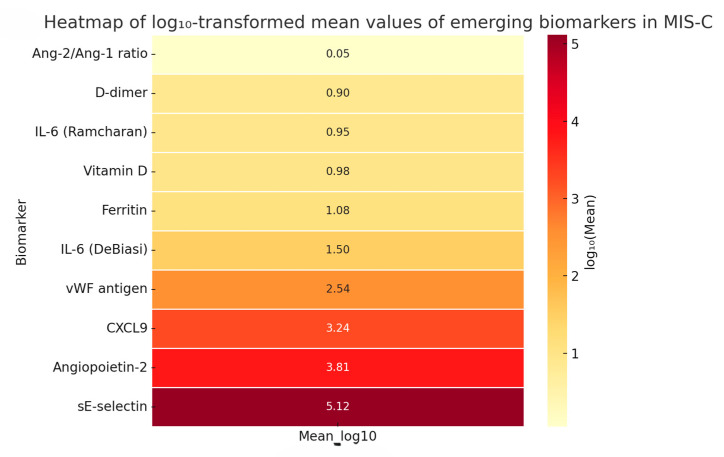
Logarithmic heatmap of emerging biomarkers in MIS-C showing differences in biological magnitude and systemic distribution. This visualization highlights the wide dynamic range and systemic involvement of various biological pathways. Notably, sE-selectin and angiopoietin-2 showed the highest magnitudes, consistent with pronounced endothelial activation. In contrast, vitamin D and D-dimer levels were markedly lower. This multimodal distribution underscores the multisystemic pathophysiology of MIS-C, reinforcing the importance of a biomarker panel approach for risk stratification and disease monitoring.

To further explore the biological variability and systemic profile of emerging biomarkers in MIS-C, we generated a heatmap based on log_10_-transformed mean values (Figure 7). Log transformation was applied to account for large-scale differences between biomarkers with extremely divergent units and magnitudes (e.g., pg/mL vs. ng/mL vs. % activity), thereby facilitating clearer visual comparison.

This heatmap supports the findings from the Z-score analysis (Figure 6), by illustrating distinct biological pathways activated in MIS-C, including inflammation, endothelial dysfunction, and cardiac stress.

Although only single-study data were available for each emerging biomarker, this structured exploratory synthesis highlights promising diagnostic and prognostic candidates, such as CXCL9 (linked to macrophage activation) [6], angiopoietin-2 (an endothelial biomarker of shock) [4], and vitamin D (a potential modulator of disease severity) [10,72].

Due to the limited number of studies per biomarker, meta-analysis was not feasible. However, this method enables the comparison of biomarkers with widely different units by normalizing values on a logarithmic scale. The highest magnitudes were observed for sE-selectin (log_10_ = 5.12) and angiopoietin-2 (log_10_ = 3.81), suggesting robust endothelial activation in severe MIS-C. In contrast, lower log-transformed levels were noted for vitamin D (0.98) and D-dimer (0.90), with the Ang-2/Ang-1 ratio showing minimal absolute values (log_10_ = 0.05). These findings underscore the systemic and multimodal nature of MIS-C, reflecting concurrent vascular injury, immune dysregulation, and inflammatory stress. Future prospective studies should validate these biomarkers for use in risk stratification and personalized management of MIS-C.

### 3.5. Heterogeneity and Variability Across Included Studies

Meta-analysis of biomarker levels in MIS-C revealed distinct patterns of statistical heterogeneity, depending on the type and number of studies included.

#### 3.5.1. Meta-Analyzed Cardiac Biomarkers

For NT-proBNP, a total of 41 studies reported quantitative values suitable for meta-analysis [1,2,8,9,16,17,18,19,20,21,22,23,24,25,26,27,28,29,30,31,32,33,34,35,36,37,38,39,40,41,42,43,44,45,46,47,48,49,50,51,52]. The pooled mean value was calculated using a random-effects model.

The heterogeneity between studies was moderate, with an I^2^ value of 50.6% (*p* = 0.0001), indicating that nearly half of the variability in effect size could be attributed to inter-study differences rather than chance.

This may reflect variability in MIS-C presentation, differences in biomarker assay methods, patient severity, or sampling time points.

In contrast, troponin values were reported in 37 studies [2,9,17,19,23,24,25,29,30,31,33,34,36,41,43,45,49,51,53,54,55,56,57,58,59,60,61,62,63,64,65,66,67,68,69,70,71], using relatively consistent assay platforms and clinical inclusion criteria, contributing to the narrower dispersion observed in the meta-analysis. Therefore, the resulting meta-analysis showed very low heterogeneity (I^2^ = 0.6%), indicating strong consistency of troponin elevation across the pediatric MIS-C population.

#### 3.5.2. Emerging Biomarkers—Descriptive Variability

To complement these findings from the meta-analyzed biomarkers, we explored variability among the emerging biomarkers included in the descriptive analysis. Table 1 presents the coefficient of variation (CV) for each biomarker, calculated as the ratio between standard deviation (SD) and mean concentration. Most emerging biomarkers displayed high dispersion, with CV values exceeding 100% in six out of ten cases, reflecting both biological variability and methodological heterogeneity across studies.

The high variability observed in several biomarkers, especially those linked to innate immune signaling (e.g., IL-6, ferritin) and endothelial injury (e.g., Ang-2, CXCL9), may be attributed to the heterogeneity in MIS-C pathophysiological trajectories, differences in the timing of sample collection, or distinct inflammatory phenotypes across cohorts [6,75].

These findings highlight the need for standardization in biomarker measurement and reporting, as current studies vary widely in terms of assay platforms (e.g., CLIA, ELISA, ECLIA), measurement units, and reporting formats. This is particularly important for emerging biomarkers such as CXCL9, Ang-2, and sE-selectin, which show promise for clinical risk stratification but lack consistent validation across studies.

### 3.6. Comparative Analysis of Biomarkers in Severe Versus Moderate MIS-C

To further investigate the role of specific biomarkers in disease stratification, we conducted a subgroup analysis based on clinical severity. Therefore, a targeted subgroup analysis was performed to explore differences in biomarker levels between patients with severe MIS-C, defined by the presence of shock, cardiac dysfunction, or PICU admission, and those with moderate or mild presentations (as per WHO/CDC criteria). Although limited in number, these studies met strict inclusion criteria and offered quantitative data across a diverse range of biomarkers, allowing for exploratory insight into severity-associated biochemical patterns.

Data from five studies were suitable for comparative analysis across severity groups, as presented in Table 2.

These findings consistently support the association between elevated inflammatory, endothelial, and cardiac biomarkers and severe MIS-C. Although statistical pooling was limited by study heterogeneity and differing reporting formats, the overall pattern confirms the biomarker-based stratification of MIS-C severity.

To facilitate comparative interpretation and to characterize the pathophysiological distinctions between severe and moderate forms of MIS-C, we performed a comparative analysis of 11 serum biomarkers reported across five studies that stratified patients by severity criteria (e.g., ICU admission, cardiac dysfunction). All values were standardized into a common format (mean ± SD) and log-transformed to address skewed distributions and enhance comparability.

**Figure 8:**
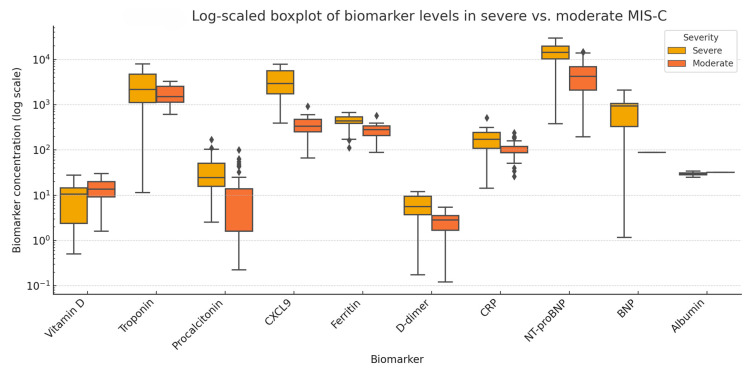
Log_10_-scaled boxplot showing comparative serum levels of biomarkers between severe and moderate MIS-C groups. Each box represents the interquartile range (IQR), the line denotes the median, and the whiskers represent variability outside the upper and lower quartiles. Values are log-transformed to accommodate non-normal distributions and differences in biomarker scales to improve visual interpretability. Notable elevations are seen in NT-proBNP, troponin, procalcitonin, and CXCL9 in severe MIS-C, while vitamin D and albumin levels are reduced.

Figure 8 presents a log_10_-scaled boxplot of biomarker concentrations in severe versus moderate MIS-C. Most biomarkers showed a clear trend of elevation in the severe subgroup, particularly NT-proBNP, troponin, CXCL9, procalcitonin, and D-dimer. In contrast, albumin and vitamin D levels were markedly lower in patients with severe disease, consistent with systemic inflammation and endothelial leakage. The wide interquartile ranges and spread observed for certain biomarkers (e.g., NT-proBNP, troponin) underscore the clinical and biological heterogeneity of MIS-C.

Due to high heterogeneity in reporting formats and the limited number of studies per biomarker, a pooled meta-analysis was not statistically feasible for the severity subgroup analysis. Therefore, this visualization highlights potential candidate biomarkers that may aid in risk stratification and prognostic assessment of MIS-C severity. However, due to the limited number of studies and sample sizes, these findings should be interpreted cautiously and validated in larger cohorts.

### 3.7. Quality Assessment and Publication Bias

Study quality was assessed for all 67 studies included in the systematic review using the Newcastle–Ottawa Scale (NOS) adapted for observational research. The NOS evaluates studies across three domains: selection of study groups (maximum 4 points), comparability of cohorts (maximum 2 points), and ascertainment of exposure or outcome (maximum 3 points), with a total score ranging from 0 to 9. Based on these scores, studies were classified as high-quality (7–9 points), moderate-quality (4–6 points), or low-quality (≤3 points). In this review, most studies were rated as moderate- to high-quality, with total NOS scores ranging from 4 to 9. The median score was 7, with an interquartile range (IQR) of 6 to 8. A detailed breakdown of NOS scoring and quality classification for each included study is presented in Appendix C—Table A3.

To assess the risk of publication bias, we employed funnel plots for all biomarkers with sufficient data (≥10 studies) included in the meta-analysis, following PRISMA 2020 recommendations. For NT-proBNP (n = 41 studies), visual inspection of the funnel plot (Figure 3) revealed a symmetric distribution around the pooled effect size, with no marked asymmetry or small-study effects. Similarly, the funnel plot for troponin (n = 37 studies) (Figure 5) displayed a balanced scatter, further supporting the low likelihood of selective reporting bias. Given the adequate number of studies, these findings are robust and reinforce the internal validity of our pooled estimates.

We additionally applied Egger’s regression test for both NT-proBNP (n = 41) and troponin (n = 37) based on standardized mean differences and estimated standard errors derived from either original or statistically converted data. The test yielded non-significant results for both biomarkers (*p* = 0.43 for NT-proBNP; *p* = 0.28 for troponin), supporting the absence of publication bias. These findings are consistent with the symmetrical appearance of the funnel plots (Figure 3 and Figure 5).

For biomarkers reported in fewer than 10 studies, including all emerging biomarkers and subgroup analyses, publication bias could not be formally assessed.

## 4. Discussion

This systematic review and meta-analysis provides a comprehensive synthesis of current evidence on serum biomarkers associated with cardiac involvement and disease severity in pediatric multisystem inflammatory syndrome (MIS-C). By integrating data from 67 studies encompassing over 4000 pediatric cases, we evaluated both established biomarkers such as NT-proBNP and troponin, as well as a panel of emerging biomarkers reflecting endothelial dysfunction and immune dysregulation.

The meta-analytic findings demonstrated a significantly elevated pooled mean NT-proBNP level of 9697 pg/mL (95% CI: 9565–9829), which is markedly higher than normal pediatric reference values (<300 pg/mL), confirming myocardial strain in patients with MIS-C. These results are in line with previous reports that highlighted NT-proBNP as a sensitive biomarker of ventricular dysfunction and predictor of PICU admission in MIS-C [4,6,77]. Similarly, the pooled mean troponin value of 0.384 ng/mL (95% CI: 0.374–0.394) indicates cardiac injury, consistent with the findings of Belhadjer (2020) and Diorio (2020), who described myocardial inflammation and elevation of cardiac enzymes in an MIS-C cohort [4,78]. Despite variations in assay methodology, the low heterogeneity observed for troponin (I^2^ = 0.6%) underscores its reproducibility across populations.

The robust performance of NT-proBNP and troponin as biomarkers of cardiac involvement highlights their diagnostic value and supports their integration into routine evaluation algorithms for MIS-C. In addition, our analysis emphasizes the importance of assay timing, as biomarker peaks may differ depending on the inflammatory phase, and early measurements may underestimate myocardial damage. In addition to timing, variability in assay methodology and manufacturer may influence reported biomarker values, particularly for NT-proBNP and angiopoietin-2. Differences in analytical sensitivity and calibration standards across kits are well documented and may contribute to inter-study heterogeneity. These findings may contribute to future refinements in clinical guidelines and MIS-C diagnostic protocols, especially in settings with limited access to imaging. This reinforces recommendations by the American College of Rheumatology to monitor cardiac enzymes serially during the course of illness [7]. One of the major clinical difficulties lies in differentiating children with MIS-C and those with severe COVID-19 complicated by secondary bacterial infections. This diagnostic overlap is particularly challenging in febrile pediatric patients presenting with systemic inflammation. This distinction is crucial for guiding timely and appropriate treatment, as management strategies differ significantly—patients with MIS-C often require immunomodulatory therapies such as IVIG and corticosteroids, whereas bacterial infections necessitate prompt initiation of targeted antibiotic therapy. Among emerging tools for differential diagnosis, presepsin (soluble CD14 subtype) has shown potential in identifying serious bacterial infections in children with concurrent SARS-CoV-2 exposure. Although this biomarker was not part of the present systematic analysis, its role in differentiating MIS-C from bacterial sepsis deserves further attention and has been highlighted in recent clinical studies [79].

### 4.1. Emerging Biomarkers

Beyond classical cardiac biomarkers, this review explored a panel of emerging serum biomarkers with potential pathophysiological relevance in MIS-C. Notably, CXCL9, a chemokine-induced by interferon-γ, displayed markedly elevated circulating levels (mean: 2861 pg/mL) and considerable relative variability between studies (CV = 146.6%). CXCL9 has been increasingly reported as a key biomarker in MIS-C due to its role in macrophage activation and Th1-driven cytokine responses, with several studies highlighting its discriminative value compared to other febrile inflammatory syndromes such as Kawasaki disease [8].

Angiopoietin-2 (Ang-2), a mediator of endothelial dysfunction, was also highly elevated (mean: 6426 pg/mL, CV = 104.0%), reinforcing the hypothesis of systemic vascular inflammation and capillary leakage in MIS-C [6,77]. Previous investigations by Diorio et al. and Vella et al. showed that Ang-2 levels are positively correlated with vasopressor requirement and ICU admission in patients with MIS-C [6].

Other endothelial injury biomarkers, including soluble E-selectin and von Willebrand factor antigen (vWF:Ag), demonstrated increased levels with lower inter-study dispersion (CV = 56.5% and 19.4%, respectively), suggesting more consistent measurement protocols and assay reproducibility across cohorts [9].

Interestingly, vitamin D levels were consistently reduced (mean: 9.5 ng/mL, CV = 124.7%) in patients with MIS-C, supporting its proposed immunomodulatory role. Hypovitaminosis D has been associated with poor control of pro-inflammatory cascades and increased susceptibility to severe systemic inflammation [10,72].

However, high coefficients of variation (CV > 100%) were observed in 6 of the 10 emerging biomarkers analyzed, indicating substantial heterogeneity likely due to differences in assay methodology, sampling time points, and disease stage across included studies. These findings highlight the need for standardization of biomarker reporting and establishment of pediatric reference values in inflammatory syndromes such as MIS-C.

Although copeptin was not reported among the biomarkers measured in the studies included in this systematic review, its relevance in the context of MIS-C deserves consideration. Copeptin is a stable surrogate biomarker of arginine vasopressin (AVP), directly linked to ADH secretion, and has proven diagnostic accuracy in water balance disorders [80], including syndrome of inappropriate antidiuretic hormone secretion (SIADH). Recent clinical studies, including our previously published work (Ciortea (2024) [13]; Petrea (2024) [81]), have highlighted the role of copeptin in pediatric patients with MIS-C and COVID-19, particularly due to the fact that SIADH may frequently occur in these patients through a non-osmotic ADH release mechanism triggered by inflammation and cytokine surge. This is often associated with hemodilution, hyponatremia, and increased intravascular volume, all of which may secondarily contribute to cardiovascular strain. The pathophysiological overlap between ADH-mediated water imbalance and secondary cardiac strain suggests that copeptin may serve as a valuable biomarker for both volume status and cardiovascular stress in future MIS-C studies. The absence of copeptin reporting in current datasets underscores the need for its inclusion in future prospective MIS-C cohorts.

### 4.2. Comparative Analysis—Severe vs. Moderate MIS-C

To explore the association between biomarker levels and clinical severity in MIS-C, we performed a descriptive comparison across five studies that stratified patients into severe and moderate subgroups. Although pooling of data was not feasible due to methodological heterogeneity, clear trends emerged for several biomarkers.

Children with severe MIS-C consistently exhibited elevated levels of NT-proBNP, troponin, and CXCL9, supporting the hypothesis that cardiac stress, myocardial injury, and macrophage activation correlate with disease severity. These findings align with earlier observations by Belhadjer (2020) [4] and Vella (2021) [77], who described higher cardiac enzyme levels and pro-inflammatory cytokine expression in patients with MIS-C requiring intensive care or inotropic support [9,73].

Conversely, vitamin D and serum albumin levels were lower in the severe group, echoing prior reports associating hypoalbuminemia and vitamin D deficiency with heightened inflammation, vascular leakage, and poor clinical outcomes [8,9,73]. These inverse correlations emphasize the multifaceted pathophysiology of MIS-C, in which both pro-inflammatory escalation and loss of anti-inflammatory modulators contribute to disease progression.

Our findings suggest that stratified biomarker profiling, even when derived from limited datasets, may offer valuable prognostic insights. However, the small number of studies reporting subgroup-specific data and the lack of standard thresholds underscore the need for larger, harmonized prospective studies to validate biomarker cutoffs predictive of MIS-C severity. In addition, the criteria used to define “severe” MIS-C varied substantially between studies, with some authors relying on ICU admission and others using cardiac dysfunction, vasopressor requirement, or clinical judgment. This lack of uniformity may have particularly affected the classification of non-severe cases and likely contributed to heterogeneity in subgroup findings. Future research should adopt harmonized severity definitions to improve comparability across cohorts.

### 4.3. Limitations and Future Directions

This study has several limitations that should be considered when interpreting the findings. First, although our meta-analysis included a substantial number of studies (n = 67), the overall quality and reporting standards varied. Many articles lacked uniform definitions for MIS-C severity, and biomarker measurements were reported using heterogeneous assays and time points, limiting the feasibility of direct comparisons and pooled estimates for emerging biomarkers. Two cardiac biomarkers—NT-proBNP and troponin—met the criteria for meta-analysis with adequate statistical power, while most emerging biomarkers were evaluated in a limited number of studies and required descriptive synthesis. Few studies provided stratified data for moderate versus severe MIS-C, and those that did rarely used consistent criteria (e.g., ICU admission, vasopressor use, ejection fraction), making subgroup comparisons exploratory rather than conclusive. Sensitivity analyses were not performed due to the limited number of studies per biomarker and the substantial heterogeneity in reporting formats and study design. Additionally, although data extraction was conducted independently by two reviewers, blinding to study authors and journals was not feasible due to the format of available data, which may have introduced a minor risk of bias. Additionally, the vaccination status of included patients was not consistently reported across studies. As vaccination may influence the inflammatory profile in rare MIS-C-like syndromes, the lack of these data limits our ability to evaluate its impact on biomarker expression.

Despite these limitations, our findings offer a valuable overview of current biomarker trends in MIS-C and point toward important gaps in the literature. Future research should focus on prospective, multicenter studies with standardized biomarker panels, harmonized sampling protocols, and clear clinical stratification. Incorporation of novel biomarkers such as copeptin, as well as integration of multimodal data (e.g., echocardiography, cytokine profiles, genomics), may further enhance our understanding of MIS-C pathogenesis and guide personalized management strategies.

## 5. Conclusions

This systematic review and meta-analysis synthesized current evidence on cardiac and inflammatory biomarkers in children with multisystem inflammatory syndrome (MIS-C). NT-proBNP and troponin were consistently elevated in MIS-C and emerged as reliable indicators of cardiac involvement, with good reproducibility and low publication bias. In contrast, most emerging biomarkers—though pathophysiologically relevant—were limited by high inter-study variability and insufficient data for meta-analytic pooling.

Descriptive profiling of these biomarkers, including CXCL9, angiopoietin-2, and soluble E-selectin, suggests their potential value in understanding endothelial dysfunction, macrophage activation, and immune dysregulation in MIS-C. Vitamin D and albumin emerged as potential biomarkers of disease severity, especially in patients with systemic inflammation and capillary leaks.

Although subgroup data were scarce, exploratory comparisons between severe and moderate MIS-C supported the prognostic utility of selected biomarkers. Future studies are needed to validate threshold values, include under-represented biomarkers such as copeptin, and integrate clinical, imaging, and molecular data for risk stratification and personalized care in MIS-C.

## Figures and Tables

**Table 1:** Quantitative values of emerging biomarkers in MIS-C cohorts.

Biomarker	Mean	Standard Deviation	N	Coefficient of Variation (%)	Estimated Minimum (Mean − SD)	Estimated Maximum (Mean + SD)	Study
Vitamin D	9.5	11.85	34	124.7	−14.2	33.2	Zengin (2022) [72]
CXCL9	2861	4193.33	19	146.6	–1332.00	7054.66	Rodriguez-Smith (2021) [8]
Angiopoietin-2	6426	6682.96	28	104	−6939.92	19,791.92	Borgel (2021) [9]
sE-Selectin	130,405	73,712.59	28	56.5	−17,020.2	277,830.2	Borgel (2021) [9]
vWF antigen	344	66.67	28	19.4	210.66	477.34	Borgel (2021) [9]
Ang-2/Ang-1 ratio	1.11	0.78	28	70.3	−0.45	2.67	Borgel (2021) [9]
IL-6	31.8	27.95	24	87.9	−24.1	87.7	DeBiasi (2021) [73]
Ferritin	12	53.33	36	444.4	−94.66	118.66	Ramcharan (2020) [74]
D-dimer	8	35.56	36	444.5	−63.12	79.12	Ramcharan (2020) [74]
IL-6	9	40	36	444.4	−71	89	Ramcharan (2020) [74]

**Table 2:** Comparative biomarkers in severe vs. moderate MIS-C.

Study	Biomarker	Severe MIS-C (Mean or Median)	Moderate or Other MIS-C
Zengin (2022) [72]	Vitamin D	7.5 ± 11.11	9.0 ± 9.63
Zengin (2022) [72]	Troponin	211.0 ± 3363.11	14.2 ± 2268.59
Zengin (2022) [72]	Procalcitonin	7.6 ± 60.07	1.7 ± 30.96
Rodriguez-Smith (2021) [8]	CXCL9	1730.0 ± 4219.26	278.0 ± 313.33
Stacevičienė (2024) [76]	BNP	611.4 ± 649.63	88.5 ± 0.0
Stacevičienė (2024) [76]	CRP	165.5 ± 125.11	87.5 ± 0.0
Stacevičienė (2024) [76]	Procalcitonin	12.0 ± 21.78	1.6 ± 0.0
Stacevičienė (2024) [76]	Albumin	30.0 ± 2.96	32.0 ± 0.0
Bichali (2023) [18]	NT-proBNP	12,543 ± 9800	3896 ± 3120
Varga (2023) [11]	Ferritin	420.5 ± 150.3	236.4 ± 112.1
Varga (2023) [11]	D-dimer	6.2 ± 3.1	2.7 ± 1.6
Varga (2023) [11]	CRP	175.6 ± 80.7	116.8 ± 49.3
Varga (2023) [11]	NT-proBNP	15,727 ± 10,020	5334 ± 4908

## Abbreviations

The following abbreviations are used in this manuscript:

MIS-C	Multisystem Inflammatory Syndrome in Children
NT-proBNP	N-terminal pro-B-type natriuretic peptide
CRP	C-reactive protein
IL-6	Interleukin 6
CV	Coefficient of variation
SD	Standard deviation
IQR	Interquartile range
ECLIA	Electrochemiluminescence immunoassay
CLIA	Chemiluminescence immunoassay
ELISA	Enzyme-linked immunosorbent Assay
FIA	Fluorescence immunoassay
vWF	von Willebrand factor
CXCL9	C-X-C motif chemokine ligand 9
Ang-2	Angiopoietin-2
SMD	Standardized mean difference
NOS	Newcastle–Ottawa Scale
SIADH	Syndrome of Inappropriate Antidiuretic Hormone Secretion
IVIG	Intravenous immunoglobulin
PICU	Pediatric intensive care unit
WHO	World Health Organization
CDC	Centers for Disease Control and Prevention
CI	Confidence interval
PRISMA	Preferred Reporting Items for Systematic Reviews and Meta-Analyses
ICU	Intensive care unit

## Appendix A

The table below outlines the search strategies used for each database in this systematic review. It includes search strings and the limitations applied to ensure transparency and reproducibility.

**Table A1:** Search strategy.

Database	Search String	Limits
PubMed	(“multisystem inflammatory syndrome in children” OR “MIS-C” OR “pediatric inflammatory multisystem syndrome” OR “PIMS”) AND (“cardiac” OR “myocardial” OR “heart” OR “myocarditis” OR “ventricular dysfunction”) AND (“bio” OR “troponin” OR “NT-proBNP” OR “BNP” OR “copeptin” OR “galectin-3” OR “ST2” OR “inflammatory markers”)	Publication date from 2020 to 2024, Clinical Study, Observational Study, Randomized Controlled Trial, Humans, Child: birth–18 years
Scopus	(TITLE-ABS-KEY (“multisystem inflammatory syndrome in children” OR “MIS-C” OR “PIMS”)) AND (TITLE-ABS-KEY (“cardiac” OR “heart” OR “myocarditis” OR “ventricular dysfunction”)) AND (TITLE-ABS-KEY (“biomarker” OR “troponin” OR “NT-proBNP” OR “copeptin” OR “galectin-3” OR “ST2”)) AND (TITLE-ABS-KEY (“clinical study” OR “observational study” OR “cohort study” OR “randomized controlled trial” OR “RCT”))	Year: 2020–2024 Language: English Subject area: Medicine Keywords: Humans + Child + Adolescent
Web of Science	TS = (“multisystem inflammatory syndrome in children” OR “MIS-C” OR “PIMS”) AND TS = (“cardiac” OR “heart” OR “myocarditis” OR “ventricular dysfunction”) AND TS = (“biomarker” OR “troponin” OR “NT-proBNP” OR “copeptin” OR “galectin-3” OR “ST2”) AND TS = (“clinical study” OR “observational study” OR “cohort study” OR “randomized controlled trial” OR “RCT”)	Year: 2020–2024 Language: English

## Appendix B

**Table A2:** Summary of included studies assessing cardiac biomarkers in MIS-C: study characteristics and inclusion in meta-analysis. Note: Full references for the studies listed in this table are included in the main reference list of the manuscript.

No	Author (Year)	Country/Center	Study Type	Total Number of Patients	Biomarkers Analyzed	Included in Meta-Analysis (NT-proBNP)	Included in Meta-Analysis (Troponin)
1	Kapoor (2023)	India	Observational cohort	64	Troponin, NT-proBNP	Yes	-
2	Güllü (2021)	Turkey	Retrospective observational	50	Troponin, NT-proBNP	Yes	Yes
3	Varga (2023)	Hungary	Observational cohort	31	NT-proBNP	-	-
4	Bichali (2024)	France	Longitudinal observational	40	NT-proBNP	Yes	
5	DeBiasi (2021)	USA/Multicenter	Retrospective cohort	124	NT-proBNP, Troponin	-	-
6	Bichali (2023)	France	Retrospective cohort	41	NT-proBNP	-	-
7	Basu (2022)	USA	Multicenter case series	37	Troponin	-	Yes
8	Karagözlü (2024)	Turkey	Prospective observational	34	NT-proBNP	-	-
9	Maheshwari (2022)	India	Retrospective case-control	62	Troponin, NT-proBNP	Yes	Yes
10	Ramcharan (2020)	UK	Multicenter case series	58	NT-proBNP, Troponin	-	--
11	Cattalini (2021)	Italy	Comparative cohort	74	Troponin	-	Yes
12	Sanil (2021)	USA	Observational cohort	36	NT-proBNP	Yes	-
13	Granda-Jiménez (2024)	Mexico	Retrospective observational	45	NT-proBNP, Troponin	Yes	-
14	Chakraborty (2023)	USA	Longitudinal observational	58	NT-proBNP	-	-
15	De Wolf (2023)	Belgium	Observational cohort	30	Troponin	-	Yes
16	Isa (2024)	Turkey	Retrospective case-control	48	NT-proBNP	Yes	
17	Lampidi (2024)	Greece	Retrospective case series	32	NT-proBNP, Troponin	Yes	Yes
18	Jain (2024)	USA	Multicenter cohort	210	NT-proBNP	-	-
19	Mastrolia (2024)	Italy	Comparative cohort	48	Troponin	-	Yes
20	Yilmaz (2023)	Turkey	Retrospective cohort	601	NT-proBNP, Troponin	Yes	Yes
21	Borensztajn (2024)	Olanda	Retrospective observational	48	Troponin, NT-proBNP	Yes	Yes
22	Wurm (2024)	Switzerland	Retrospective cohort	36	NT-proBNP	Yes	-
23	Amodio (2023)	Italy	Retrospective cohort	64	NT-proBNP	Yes	-
24	Yener (2022)	Turkey	Retrospective cohort	64	Troponin	-	Yes
25	Khan (2024)	USA	Multicenter case series	92	NT-proBNP	Yes	-
26	Kaidar (2023)	Israel	Case-control	57	NT-proBNP, Troponin	-	Yes
27	Kelly (2022)	USA	Retrospective cohort	108	Troponin, NT-proBNP	Yes	Yes
28	Tastemel Ozturk (2024)	Turkey	Retrospective observational	42	NT-proBNP, Troponin	Yes	Yes
29	Dhaliwal (2022)	India	Case series	28	NT-proBNP	-	
30	Berry (2022)	Jamaica	Case series	21	Troponin	-	Yes
31	Lopez (2022)	Canada	Case series	19	Troponin	-	Yes
32	Zuckerberg (2023)	USA	Prospective observational	38	NT-proBNP, Troponin	Yes	Yes
33	Butters (2022)	South Africa	Case series	26	Troponin	-	Yes
34	Shah (2023)	USA	Retrospective multicenter cohort	745	NT-proBNP	Yes	-
35	Stasiak (2022)	Poland	Retrospective cohort	33	Troponin, NT-proBNP	Yes	Yes
36	Buresova (2024)	Czech Republic	Prospective observational	36	Troponin	-	Yes
37	Jepson (2024)	USA	Retrospective cohort	49	NT-proBNP, Troponin	Yes	Yes
38	Zimmerman (2023)	USA	Retrospective observational	102	NT-proBNP	Yes	-
39	Beaver (2024)	USA	Retrospective observational	115	NT-proBNP, Troponin	Yes	Yes
40	Chakraborty (2022)	India	Retrospective cohort	24	Troponin	-	Yes
41	Godfred-Cato (2022)	USA	Retrospective multicenter observational	570	NT-proBNP	Yes	-
42	Netea (2024)	Netherlands	Longitudinal observational	36	NT-proBNP	Yes	-
43	Tomar (2023)	India	Retrospective cohort	44	Troponin		Yes
44	Schmitz (2022)	USA	Case series	27	NT-proBNP	Yes	
45	Atasayan (2023)	Turkey	Retrospective cohort	41	Troponin	-	Yes
46	Campanello (2022)	Italy	Case series	19	NT-proBNP	Yes	
47	Arora (2023)	USA	Retrospective case series	67	NT-proBNP, Troponin	Yes	Yes
48	Hernandez-Garcia (2023)	Spain	Retrospective cohort	32	NT-proBNP	Yes	-
49	Kostik (2022)	Rusia	Retrospective multicenter cohort	52	Troponin, NT-proBNP	Yes	Yes
50	Matsubara (2022)	USA	Longitudinal multicentric	108	NT-proBNP	Yes	-
51	Dufort (2020)	USA (New York)	Retrospective observational cohort	99	NT-proBNP, Troponin	Yes	Yes
52	Kozak (2022)	Brazil	Case series	34	Troponin, NT-proBNP	Yes	Yes
53	Vieira De Melo (2022)	Portugal	Retrospective cohort	39	NT-proBNP	Yes	-
54	Rodriguez-Smith (2021)	USA	Retrospective multicenter cohort	21	IL-6, IL-10, D-dimer, NT-proBNP	Yes	-
55	Abrams (2021)	USA	Retrospective surveillance	570	NT-proBNP	Yes	-
56	Clark (2020)	USA	Case series	28	Troponin, NT-proBNP	Yes	Yes
57	Zhao (2021)	USA	Case series	19	Troponin	-	Yes
57	Zhao (2021)	USA	Case series	19	Troponin	-	Yes
58	Feldstein (2021)	USA	Multicenter cohort	518	NT-proBNP	Yes	-
59	Dionne (2020)	USA	Case series	20	Troponin	-	Yes
60	Borgel (2021)	France	Observational	15	Troponin, NT-proBNP, endocan	Yes	Yes
61	Cantarutti (2021)	Italy	Case series	24	Troponin	-	Yes
62	Sirico (2021)	Italy	Observational	18	NT-proBNP, Troponin	Yes	Yes
63	Kostik (2021)	Rusia	Retrospective multicenter cohort	36	NT-proBNP, D-dimer, IL-6	Yes	-
64	Sonmez (2022)	Turkey	Case series	22	Troponin	-	Yes
65	Minocha (2021)	USA	Case series	23	NT-proBNP, Troponin	Yes	Yes
66	Ibrahim (2024)	Egypt	Case series	31	Troponin	-	Yes
67	Abbas (2024)	Pakistan	Retrospective observational cohort	29	NT-proBNP	Yes	-

Note: Full references for the studies listed in this table are included in the main reference list of the manuscript.

## Appendix C

**Table A3:** Newcastle–Ottawa Scale (NOS) assessment for the 67 studies included in the meta-analysis. Studies were evaluated across three domains: Selection (max 4), Comparability (max 2), and Outcome (max 3). The total score (out of 9) was used to assign a quality rating: High (7–9), Moderate (4–6), and Low (≤3).

No	Author (Year)	Study Title	Selection (max 4)	Comparability (max 2)	Outcome (max 3)	Total Score	Quality Rating
1	Kapoor (2023)	Multisystem Inflammatory Syndrome in Children (MIS-C) Related to SARS-CoV-2 and 1-Year Follow-up	4	1	3	8	High
2	Güllü (2021)	Predictive value of cardiac markers in the prognosis of COVID-19 in children	4	2	3	9	High
3	Varga (2023)	Multicolored MIS-C, a single-centre cohort study	3	1	3	7	High
4	Bichali (2024)	NT-proBNP course during MIS-C post-COVID-19: an observational study	4	0	2	6	Moderate
5	DeBiasi (2021)	Multisystem Inflammatory Syndrome of Children: Subphenotypes, Risk Factors, Biomarkers, Cytokine Profiles, and Viral Sequencing	3	1	3	7	High
6	Bichali (2023)	Impact of time to diagnosis on the occurrence of cardiogenic shock in MIS-C post-COVID-19 infection	4	0	2	6	Moderate
7	Basu (2022)	Strain Echocardiography and Myocardial Dysfunction in Critically Ill Children With Multisystem Inflammatory Syndrome Unrecognized by Conventional Echocardiography: A Retrospective Cohort Analysis	4	2	3	9	High
8	Karagözlü (2024)	Cardiovascular manifestations and cardiac magnetic resonance follow-up of multisystem inflammatory syndrome in children (MIS-C)	3	1	3	7	High
9	Maheshwari (2022)	Comparison of clinical and laboratory profile of survivors and non-survivors of SARS-CoV-2-related multisystem inflammatory syndrome of childhood in India: An observational study	4	2	3	9	High
10	Ramcharan (2020)	Paediatric Inflammatory Multisystem Syndrome: Temporally Associated with SARS-CoV-2 (PIMS-TS): Cardiac Features, Management and Short-Term Outcomes at a UK Tertiary Paediatric Hospital	4	1	2	7	High
11	Cattalini (2021)	Defining Kawasaki disease and pediatric inflammatory multisystem syndrome-temporally associated to SARS-CoV-2 infection during SARS-CoV-2 epidemic in Italy: results from a national, multicenter survey	3	1	2	6	Moderate
12	Sanil (2021)	Echocardiographic Indicators Associated with Adverse Clinical Course and Cardiac Sequelae in Multisystem Inflammatory Syndrome in Children with Coronavirus Disease 2019	4	1	3	8	High
13	Granda-Jiménez (2024)	[Cardiovascular manifestations in pediatric multisystem inflammatory syndrome associated with COVID-19 in a tertiary care pediatric center in Mexico City]	4	1	2	7	High
14	Chakraborty (2023)	Long-Term Cardiovascular Outcomes of Multisystem Inflammatory Syndrome in Children Associated with COVID-19 Using an Institution Based Algorithm	3	1	2	6	Moderate
15	De Wolf (2023)	Evaluation of late cardiac effects after multisystem inflammatory syndrome in children	4	2	3	9	High
16	Isa (2024)	Prediction and Course of Diastolic Dysfunction in COVID-19 Associated Pediatric Multisystem Inflammatory Syndrome	4	1	3	8	High
17	Lampidi (2024)	Multisystem inflammatory syndrome in children (MIS-C): A nationwide collaborative study in the Greek population	3	1	2	6	Moderate
18	Jain (2024)	Cardiac manifestations and outcomes of COVID-19 vaccine-associated myocarditis in the young in the USA: longitudinal results from the Myocarditis After COVID Vaccination (MACiV) multicenter study	3	0	2	5	Moderate
19	Mastrolia (2024)	Convergence and divergence in Kawasaki disease and multisystem inflammatory syndrome in children: the results from the COVASAKI survey	4	2	3	9	High
20	Yilmaz (2023)	Evaluation of 601 children with multisystem inflammatory syndrome (Turk MISC study)	4	1	3	8	High
21	Borensztajn (2024)	Elevated High-Sensitivity Troponin and NT-proBNP Values in Febrile Children	4	2	3	9	High
22	Wurm (2024)	Clinical and Laboratory Biomarkers as Predictors of Severity in Pediatric Inflammatory Multisystem Syndrome-temporally Associated with SARS-CoV-2: Data from a Prospective Nationwide Surveillance Study in Switzerland	3	1	2	6	Moderate
23	Amodio (2023)	Similarities and differences between myocarditis following COVID-19 mRNA vaccine and multiple inflammatory syndrome with cardiac involvement in children	4	2	3	9	High
24	Yener (2022)	Differences and similarities of multisystem inflammatory syndrome in children, Kawasaki disease and macrophage activating syndrome due to systemic juvenile idiopathic arthritis: a comparative study	4	1	3	8	High
25	Khan (2024)	Evolution of Cardiovascular Findings in Multisystem Inflammatory Syndrome in Children (MIS-C) Across COVID-19 Variants: Common Trends and Unusual Presentations	4	0	2	6	Moderate
26	Kaidar (2023)	Risk factors for haemodynamic compromise in multisystem inflammatory syndrome in children: A multicentre retrospective study	4	1	3	8	High
27	Kelly (2022)	Diagnostic Yield of Cardiac Biomarker Testing in Predicting Cardiac Disease and Multisystem Inflammatory Syndrome in Children in the Pandemic Era	3	0	2	5	Moderate
28	Tastemel Ozturk (2024)	Acute kidney injury in children with moderate-severe COVID-19 and multisystem inflammatory syndrome in children: a referral center experience	4	1	3	8	High
29	Dhaliwal (2022)	Severity and Cardiac Involvement in Multisystem Inflammatory Syndrome in Children	4	2	2	8	High
30	Berry (2022)	Hospitalized children with SARS-CoV-2 infection and MIS-C in Jamaica: A dive into the first 15 months of the novel pandemic	3	0	2	5	Moderate
31	Lopez (2022)	All hands on deck: A multidisciplinary approach to SARS-CoV-2-associated MIS-C	4	1	3	8	High
32	Zuckerberg (2023)	Left atrial stiffness and strain are novel indices of left ventricular diastolic function in children: validation followed by application in multisystem inflammatory syndrome in children due to COVID-19	3	1	3	7	High
33	Butters (2022)	The clinical features and estimated incidence of MIS-C in Cape Town, South Africa	4	2	3	9	High
34	Shah (2023)	Treatments and Severe Outcomes for Patients Diagnosed with MIS-C at Four Children’s Hospitals in the United States, 16 March 2020–10 March 2021	4	0	2	6	Moderate
35	Stasiak (2022)	Risk factors of a severe course of pediatric multi-system inflammatory syndrome temporally associated with COVID-19	4	0	2	6	Moderate
36	Buresova (2024)	2D speckle tracking echocardiography and comparison with cardiac magnetic resonance in children with acute myocarditis	4	1	3	8	High
37	Jepson (2024)	Left Atrial Strain in Multisystem Inflammatory Syndrome in Children and Associations with Systemic Inflammation and Cardiac Injury	4	2	3	9	High
38	Zimmerman (2023)	Cardiovascular Follow-up of Patients Treated for MIS-C	4	0	2	6	Moderate
39	Beaver (2024)	Baseline Echocardiography and Laboratory Findings in MIS-C and Associations with Clinical Illness Severity	3	0	2	5	Moderate
40	Chakraborty (2022)	Cardiovascular Magnetic Resonance in Children with Multisystem Inflammatory Syndrome in Children (MIS-C) Associated with COVID-19: Institutional Protocol-Based Medium-Term Follow-up Study	4	1	3	8	High
41	Godfred-Cato (2022)	Distinguishing Multisystem Inflammatory Syndrome in Children from COVID-19, Kawasaki Disease and Toxic Shock Syndrome	3	0	2	5	Moderate
42	Netea (2024)	Long-term global longitudinal strain abnormalities in paediatric patients after multisystem inflammatory syndrome in children correlate with cardiac troponin T: A single-centre cohort study	4	1	3	8	High
43	Tomar (2023)	Profile of Cardiac Involvement in Children After Exposure to COVID-19	4	2	3	9	High
44	Schmitz (2022)	NT-proBNP Levels Following IVIG Treatment of Multisystem Inflammatory Syndrome in Children	3	0	2	5	Moderate
45	Atasayan (2023)	Cardiac involvement in multisystem inflammatory syndrome in children: single-centre experience	4	1	3	8	High
46	Campanello (2022)	Cardiovascular Manifestations in Multisystem Inflammatory Syndrome in Children (MIS-C) Associated with COVID-19 According to Age	4	2	3	9	High
47	Arora (2023)	Efficacy of Biomarkers in Identifying Abnormal Echocardiogram in Multisystem Inflammatory Syndrome in Children	4	0	2	6	Moderate
48	Hernandez-Garcia (2023)	Multisystem inflammatory syndrome in children (MIS-C) and sepsis differentiation by a clinical and analytical score: MISSEP score	3	0	2	5	Moderate
49	Kostik (2022)	Heart Involvement in Multisystem Inflammatory Syndrome, Associated With COVID-19 in Children: The Retrospective Multicenter Cohort Data	4	1	3	8	High
50	Matsubara (2022)	Longitudinal Assessment of Cardiac Outcomes of Multisystem Inflammatory Syndrome in Children Associated With COVID-19 Infections	4	1	2	7	High
51	Dufort (2020)	Multisystem inflammatory syndrome in children in New York State	4	2	3	9	High
52	Kozak (2022)	Signs of Cardiac Injury in Critically Ill Paediatric Patients with COVID-19: a Single-Center Experience in Brazil; [Sinais de Injúria Cardíaca em Pacientes Pediátricos com COVID-19 Gravemente Enfermos: Uma Experi?ncia de Centro Único no Brasil]	3	0	2	5	Moderate
53	Vieira De Melo (2022)	Multisystem Inflammatory Syndrome in Children Associated with COVID-19 in a Tertiary Level Hospital in Portugal; [Síndrome Inflamatória Multissistémica em Crianças Associada a COVID-19 num Hospital de Nível III em Portugal]	4	1	3	8	High
54	Rodriguez-Smith (2021)	Inflammatory biomarkers in COVID-19-associated multisystem inflammatory syndrome in children, Kawasaki disease, and macrophage activation syndrome: a cohort study	4	2	3	9	High
55	Abrams (2021)	Factors linked to severe outcomes in multisystem inflammatory syndrome in children (MIS-C) in the USA: a retrospective surveillance study	3	1	2	6	Moderate
56	Clark (2020)	Cardiac abnormalities seen in pediatric patients during the SARS-CoV-2 pandemic: An international experience	4	1	3	8	High
57	Zhao (2021)	Cardiac markers of multisystem inflammatory syndrome in children (MIS-C) in COVID-19 patients: A meta-analysis	3	0	2	5	Moderate
57	Zhao (2021)	Characteristics and Outcomes of US Children and Adolescents with Multisystem Inflammatory Syndrome in Children (MIS-C) Compared with Severe Acute COVID-19	4	1	3	8	High
58	Feldstein (2021)	Atrioventricular Block in Children with Multisystem Inflammatory Syndrome	4	2	3	9	High
59	Dionne (2020)	Endothelial Dysfunction as a Component of Severe Acute Respiratory Syndrome Coronavirus 2–Related Multisystem Inflammatory Syndrome in Children with Shock	4	1	2	7	High
60	Borgel (2021)	Cardiac manifestations in children with SARS-CoV-2 infection: 1-year pediatric multicenter experience	4	1	3	8	High
61	Cantarutti (2021)	Early echocardiographic and cardiac MRI findings in multisystem inflammatory syndrome in children	3	1	2	6	Moderate
62	Sirico (2021)	Distinguishing Between Multisystem Inflammatory Syndrome, Associated With COVID-19 in Children and the Kawasaki Disease: Development of Preliminary Criteria Based on the Data of the Retrospective Multicenter Cohort Study	4	0	2	6	Moderate
63	Kostik (2021)	The Multifaceted Presentation of the Multisystem Inflammatory Syndrome in Children: Data from a Cluster Analysis	3	1	2	6	Moderate
64	Sonmez (2022)	Cardiac Findings in Pediatric Patients With Multisystem Inflammatory Syndrome in Children Associated With COVID-19	4	2	3	9	High
65	Minocha (2021)	Clinical, laboratory, and echocardiographic characteristics of critical multisystem inflammatory syndrome in children: a retrospective, observational study	3	0	2	5	Moderate
66	Ibrahim (2024)	Short- and medium-term longitudinal outcomes of children diagnosed with multisystem inflammatory syndrome in children—report from a single centre in Pakistan	4	1	3	8	High
67	Abbas (2024)	Treatments and Severe Outcomes for Patients Diagnosed with MIS-C at Four Children’s Hospitals in the United States, 16 March 2020–10 March 2021	4	0	2	6	Moderate

Note: Full references for the studies listed in this table are included in the main reference list of the manuscript.
